# Structures and Dissolution Behaviors of Quaternary CaO-SrO-P_2_O_5_-TiO_2_ Glasses

**DOI:** 10.3390/ma14071736

**Published:** 2021-04-01

**Authors:** Sungho Lee, Fukue Nagata, Katsuya Kato, Takayoshi Nakano, Toshihiro Kasuga

**Affiliations:** 1 National Institute of Advanced Industrial Science and Technology (AIST), 2266-98 Anagahora, Shimoshidami, Moriyama-ku, Nagoya 463-8560, Japan; f.nagata@aist.go.jp (F.N.); katsuya-kato@aist.go.jp (K.K.); 2 Division of Materials and Manufacturing Science, Graduate School of Engineering, Osaka University, 2-1 Yamadaoka, Suita, Osaka 565-0871, Japan; nakano@mat.eng.osaka-u.ac.jp; 3 Division of Advanced Ceramics, Graduate School of Engineering, Nagoya Institute of Technology, Gokiso-cho, Showa-ku, Nagoya 466-8555, Japan; kasuga.toshihiro@nitech.ac.jp

**Keywords:** bioactive glass, phosphate glass, structure, dissolution behavior, strontium

## Abstract

Calcium phosphate glasses have a high potential for use as biomaterials because their composition is similar to that of the mineral phase of bone. Phosphate glasses can dissolve completely in aqueous solution and can contain various elements owing to their acidity. Thus, the glass can be a candidate for therapeutic ion carriers. Recently, we focused on the effect of strontium ions for bone formation, which exhibited dual effects of stimulating bone formation and inhibiting bone resorption. However, large amounts of strontium ions may induce a cytotoxic effect, and there is a need to control their releasing amount. This work reports fundamental data for designing quaternary CaO-SrO-P_2_O_5_-TiO_2_ glasses with pyro- and meta-phosphate compositions to control strontium ion-releasing behavior. The glasses were prepared by substituting CaO by SrO using the melt-quenching method. The SrO/CaO mixed composition exhibited a mixed cation effect on the glassification degree and ion-releasing behavior, which showed non-linear properties with mixed cation compositions of the glasses. Sr^2+^ ions have smaller field strength than Ca^2+^ ions, and the glass network structure may be weakened by the substitution of CaO by SrO. However, glassification degree and chemical durability of pyro- and meta-phosphate glasses increased with substituted all CaO by SrO. This is because titanium groups in the glasses are closely related to their glass network structure by SrO substitution. The P-O-Ti bonds in pyrophosphate glass series and TiO_4_ tetrahedra in metaphosphate glass series increased with substitution by SrO. The titanium groups in the glasses were crosslink and/or coordinate phosphate groups to improve glassification degree and chemical durability. Sr^2+^ ion releasing amount of pyrophosphate glasses with >83% SrO substitution was larger than 0.1 mM at day seven, an amount that reported enhanced bone formation by stimulation of osteogenic markers.

## 1. Introduction

Therapeutic inorganic ions such as silicate, phosphate, magnesium, calcium, and strontium ions have been reported to stimulate tissue regeneration [1,2]. Silicate ions have been reported to improve the proliferation and differentiation of osteoblasts by stimulating insulin-like growth factor II (IGF-II) [3], alkaline phosphatase (ALP), and osteocalcin [4]. Phosphate ions are the main components of bones and stimulate the expression of matrix Gla protein to improve bone formation [5]. Magnesium ions enhance osteoblast adhesion, proliferation, differentiation, and calcification [6]. Calcium ions upregulate the expression of IGF-I and IGF-II [7] and stimulate the formation of extracellular matrix for calcification [8]. Strontium ions have two main effects: the stimulation of osteoblast proliferation and differentiation and the inhibition of preosteoclast differentiation [9,10,11]. The ions contribute to an improvement in cell proliferation by upregulating c-fos and egr-1, promoting osteoblast metabolism by activating calcium-sensing receptors, and stimulating ALP and osteopontin, which are markers for osteoblast differentiation [7,11,12,13,14]. In addition, strontium ions downregulate receptor activators of nuclear factor kappa B (RANK) ligand expression, which relates to the differentiation of pre-osteoblasts by upregulation of osteoprotegerin (OPG; known as an osteoclastogenesis inhibitory factor) [10]. The strontium-substituted hydroxyapatite scaffolds with an 0.05 of Sr/(Ca + Sr) molar ratio enhanced cell proliferation, ALP activity, calcification, and OPG. By contrast, the scaffold with the ratio of 0.1 showed cytotoxic effect on mouse osteoblastic cells (MC3T3-E1) [15]. Moghanian et al. reported that 58S bioactive glasses containing 5 mol% SrO exhibited stimulation of proliferation and ALP activity on MC3T3-E1 cells, whereas the glass containing 10 mol% SrO exhibited cytotoxic effect on the cells [16]. Strontium-containing 45S5 bioglasses were upregulated osteogenic markers in vitro, such as bone morphogenetic protein-2, osteopontin, bone sialoprotein, and osteocalcin, and enhanced bone formation in vivo [17]. Accordingly, strontium ions for biomedical applications may need to control their amount without cytotoxicity.

Calcium phosphate glasses have high potential for use as biomaterials because their composition is similar to that of the mineral phase of bone. Phosphate glasses can dissolve completely in an aqueous solution [18,19] and can contain more elements than silicate glass owing to their acidity [20]. Accordingly, the glass can be a candidate for a therapeutic ion carrier. However, phosphate glass has comparably high solubility and may induce inhibition of cell growth [21]. Thus, the solubility of phosphate glasses must be controlled for biomedical applications. Titanium dioxide is known to improve the chemical durability of phosphate glasses because Ti^4+^ ions improve the cross-linking of phosphate groups [22,23]. CaO-P_2_O_5_-TiO_2_ invert glass (denoted by PIG) has been the focus of research in our group [24,25]. PIG is composed of short phosphate groups, such as ortho- (*Q_P_*^0^) and pyrophosphates (*Q_P_*^1^) [24]. Titanium dioxide in PIG forms P-O-Ti bonds, resulting in enhanced glass-forming ability and chemical durability [25]. In our previous works, we demonstrated that phosphate invert glasses contained various therapeutic ions, such as Mg^2+^ [26,27,28], Sr^2+^ [29,30], Zn^2+^ [31], Ag^+^ [32] and Nb^5+^ [33,34] ions, and controlled their release behavior. Zinc- and niobium-containing phosphate invert glasses exhibit high chemical durability because these ions improve the cross-linking of phosphate groups [25,31]. In the case of magnesium-containing phosphate invert glasses, Mg^2+^ ions formed P-O-Mg bonds and crosslinked phosphate groups. However, the bonds were easily hydrolyzed in aqueous solutions; accordingly, magnesium-containing phosphate invert glasses had smaller chemical durability [35]. Strontium ions in phosphate invert glasses decrease chemical durability owing to their smaller field strength [29]. The ion-releasing behaviors of the phosphate invert glasses are strongly related to the glass network structure. Thus, investigating the glass structure is necessary to control ion-releasing behavior, which can improve the therapeutic activity of phosphate glasses.

The present work represents a fundamental study of strontium ions for designing novel bioactive phosphate glasses with controlled release of therapeutic ions. Strontium ions have dual effects on osteoblasts and osteoclasts and are used for bone fracture in patients with osteoporosis [36,37]. In our previous work, Na_2_O-CaO-SrO-P_2_O_5_-TiO_2_ and MgO-CaO-SrO-P_2_O_5_-TiO_2_ quinary invert glasses reported an ion-releasing amount of approximately 6% at day 7 [29,30]. Recently, our group focused on radio frequency magnetron sputtering (RF-sputtering) for the preparation of coating layer using phosphate invert glass target [38]. The coating layer exhibited comparably larger chemical durability, and the dissolution rate was 10 nm/day. However, metaphosphate groups were found in the coating layer, where the groups were not existed in the target glass. The metaphosphate groups in the coating layer may induce decreasing chemical durability, since metaphosphate groups easily hydrolyze compared to short phosphate groups [39], such as pyro- and orthophosphate. Thus, the target glass may need to improve chemical durability, using a RF-sputtering method. In this work, we prepared CaO-SrO-P_2_O_5_-TiO_2_ quaternary glasses with pyro- and meta-phosphate compositions, where Na_2_O and MgO in quinary glass systems were substituted by TiO_2_ for further improvement of chemical durability. The structures and ion-releasing behaviors of CaO-SrO-P_2_O_5_-TiO_2_ quaternary glasses were evaluated.

## 2. Materials and Methods

### 2.1. Preparation of CaO-SrO-P_2_O_5_-TiO_2_ Glasses

Two phosphate glass series were prepared wherein CaO was substituted by SrO with nominal compositions of (60−*a*)CaO·*a*SrO·30P_2_O_5_·10TiO_2_ (mol%, denoted by PIG-*x*Sr) and (45−*b*)CaO·*b*SrO·50P_2_O_5_·5TiO_2_ (mol%, denoted by MPG-*y*Sr), as listed in Table 1. Calcium carbonate (CaCO_3_), strontium carbonate (SrCO_3_), phosphoric acid (H_3_PO_4_, liquid), and titanium oxide (TiO_2_) were used for preparing the batch mixtures, and all reagents were purchased from Kishida Chemical Co., Osaka, Japan. The reagents were mixed in distilled water to produce a slurry, and the mixtures were dried at 140 °C overnight. The resulting products for the PIG-*x*Sr and MPG-*y*Sr series were melted in a platinum crucible at 1500 and 1400 °C, respectively, for 30 min. The melts were poured onto a stainless-steel plate and pressed with another stainless-steel plate for quenching. The crystalline and opaque parts were removed manually, and optically clear PIG-*x*Sr and MPG-*y*Sr were obtained.

### 2.2. Characterization of CaO-SrO-P_2_O_5_-TiO_2_ Glass Structures

The densities of PIG-*x*Sr and MPG-*y*Sr were measured by an Archimedes method with glass pieces of 500–1000 mg using water as an immersion fluid. Oxygen density, which is the index of the compactness of the glass network, was calculated using the following equation [40,41]:

(1) ρoxygen=MO×CaO+SrO+5P2O5+2TiO2Mglassρglass

where $M_{O}$ is the atomic weight of oxygen, $M_{glass}$ is the molar weight of PIG-*x*Sr or MPG-*y*Sr. [CaO], [SrO], [P_2_O_5_], and [TiO_2_] are the molar fractions of calcium oxide, strontium oxide, phosphate, and titanium dioxide, respectively, and $\rho_{glass}$ is the experimental density of PIG-*x*Sr or MPG-*y*Sr.

The glass structures of PIG-*x*Sr and MPG-*y*Sr were evaluated using laser Raman spectroscopy in the region of Raman shifts between 550 and 1400 cm^−1^ (NRS-2000, JASCO Co., Tokyo, Japan). The glasses were excited at 514.5 nm by an Ar laser with a power of 20 mW and exposure time of 30 s with an accumulation number of 16. The obtained Raman spectra of PIG-*x*Sr and MPG-*y*Sr were deconvoluted by assuming Gaussian spectral lines for quantitative analysis using a multipeak fitting package (Igor Pro 8, WaveMetrics Inc., Portland, OR, USA).

### 2.3. Thermal Analysis of CaO-SrO-P_2_O_5_-TiO_2_ Glasses

The thermal properties of PIG-*x*Sr and MPG-*y*Sr were evaluated using differential thermal analysis (DTA, Thermo plus TG8120, 5 K/min, Rigaku Co., Tokyo, Japan). The glass transition (*T_g_*) and onset of crystallization (*T_c_*) temperatures of PIG-*x*Sr and MPG-*y*Sr were obtained from the DTA traces. The glassification degree (*GD*) was calculated using the following equation [42,43]:

(2) $$GD=\;\frac{T_{c}-T_{g}}{T_{c}}\;\left[\frac{K}{K}\right]$$

and it was used as an index of ability for glass-forming ability.

### 2.4. Dissolution Behavior of CaO-SrO-P_2_O_5_-TiO_2_ Glasses

PIG-*x*Sr and MPG-*y*Sr were pulverized and sieved to a particle size of 125–250 μm to investigate their ion-releasing behavior. A 50 mM Tris-HCl solution (TBS) was prepared by dissolving tris(hydroxymethyl)aminomethane (NH_2_C(CH_2_OH)_3_, Kishida Chemical Co., Osaka, Japan) in distilled water and adjusting the pH to 7.4 with 1M HCl at 37 °C. Fifteen milligrams of the glass powder was soaked in 15 mL of TBS for 7 days at 37 °C. The concentrations of Ca^2+^, Sr^2+^, phosphate, and Ti^4+^ ions in the TBS were measured using inductively coupled plasma-atomic emission spectroscopy (ICP-AES, ICPS-7000, Shimadzu Co., Kyoto, Japan). The molar releasing fraction of each ion was calculated using the following equation [44,45]:

(3) $$Release\;precentage\;\left(\%\right)=\;\frac{\left(\frac{C_{ion}}{M_{w.atom}}\right)\times 10^{5}}{\left(\frac{Frac_{mol}\times M_{w.glass}}{m_{glass}\times V_{solution}}\right)}$$

where $C_{ion}$ (mg/L) is the concentration of the ion of interest, $M_{w.atom}\;\left(g\right)$ is the atomic weight of the respective element, $Frac_{mol}$ is the nominal molar fraction of the ions in the glass, $M_{w.glass}\;\left(g\right)$ is the molar weight of the glass, $m_{glass}\;\left(g\right)$ is the mass of the soaked glass powder, and $V_{solution}\;\left(L\right)$ is the volume of TBS.

## 3. Results

**Figure 1:**
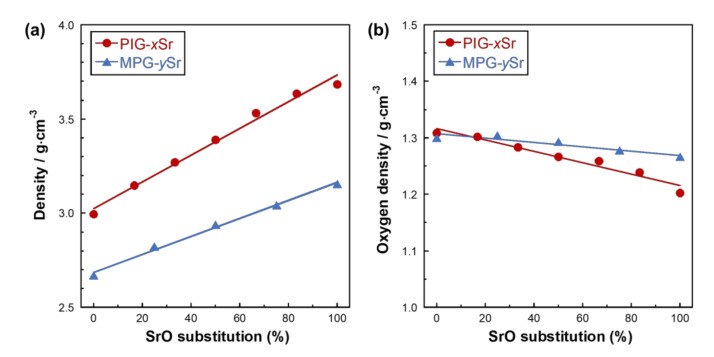
(**a**) Densities and (**b**) calculated oxygen densities of PIG-*x*Sr (circle) and (**b**) MPG-*y*Sr (triangle) as a function of SrO substitution percentage.

Figure 1 shows the density and oxygen density of PIG-*x*Sr and MPG-*y*Sr. The densities of PIG-*x*Sr and MPG-*y*Sr increased from 3.00 to 3.69 and from 2.67 to 3.16 g·cm^−3^, respectively, with increase in SrO substitution percentage. The oxygen densities of PIG-*x*Sr and MPG-*y*Sr decreased from 1.31 to 1.20 and from 1.30 to 1.27 g·cm^−3^, respectively, with an increase in SrO substitution percentage.

**Figure 2:**
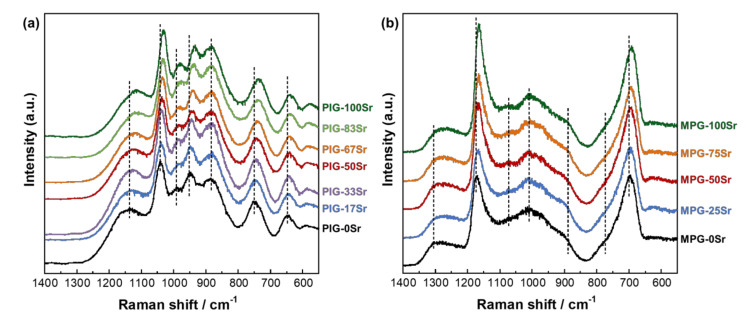
Laser Raman spectra for (**a**) PIG-*x*Sr and (**b**) MPG-*y*Sr.

The Raman spectra and the band and peak assignments of PIG-*x*Sr and MPG-*y*Sr are presented in Figure 2 and Table 2, respectively. PIG-*x*Sr exhibited Raman bands corresponding to the phosphate *Q_P_*^0^ and *Q_P_*^1^ groups [35,46,47], TiO_4_ tetrahedra, and TiO_6_ octahedra [24,48]. MPG-*y*Sr exhibited Raman bands corresponding to the phosphate *Q_P_*^1^ and *Q_P_*^2^ groups [35,46,49,50] and TiO_4_ tetrahedra [24,48].

The Raman spectra of PIG-*x*Sr and MPG-*y*Sr were simulated by assuming Gaussian lines for quantitative analysis. In the case of MPG-*y*Sr, the Raman band corresponding to the POP symmetric stretching mode of bridging oxygen (700 cm^−1^) was deconvoluted as *Q_P_*^2^ long/short chains and *Q_P_*^1^ [51], and the band corresponding to the PO_2_ symmetric stretching mode of non-bridging oxygen (1170 cm^−1^) was deconvoluted into *Q_P_*^2^ long and short chains [51]. The peaks of the phosphate groups in PIG-*x*Sr were red-shifted on increasing the SrO substitution percentage. The TiO_6_ octahedra and P-O-Ti bonds were red-shifted, whereas the positions of TiO_4_ were not significantly different from that after SrO substitution. The peaks of the phosphate and TiO_4_ groups in MPG-*y*Sr were also red-shifted when the SrO substitution percentage increased.

**Figure 3:**
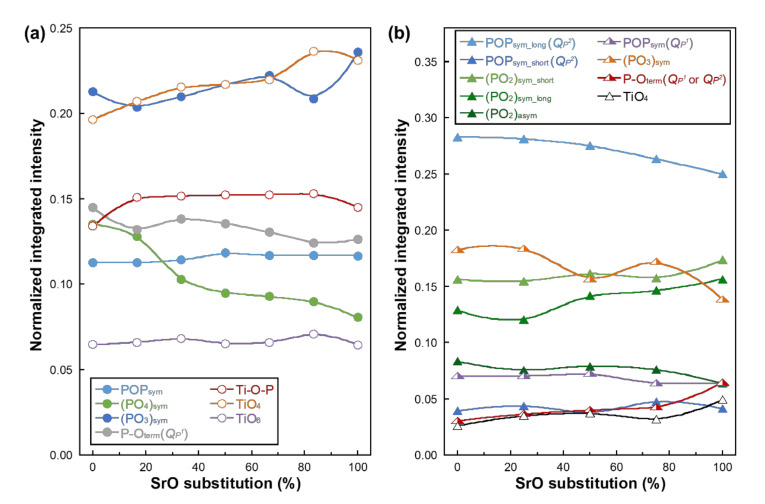
Normalized integrated peak intensities of (**a**) PIG-*x*Sr (circle) and (**b**) MPG-*y*Sr (triangle) as a function of SrO substitution percentage.

The integrated peak intensities of the deconvoluted bands were normalized by the sum of *I*(TiO_6_) + *I*(POP_sym_) + *I*(TiO_4_) + *I*((PO_4_)_sym_) + *I*(P-O-Ti) + *I*((PO_3_)_sym_) + *I*(P-O_term_(*Q_P_*^1^)) and *I*(POP_sym_long_(*Q_P_*^2^)) + *I*(POP_sym_short_(*Q_P_*^2^)) + *I*(POP_sym_(*Q_P_*^1^)) + *I*(TiO_4_) + *I*((PO_3_)_sym_) + *I*(P-O_term_(*Q_P_*^1^ or *Q_P_*^2^)) + *I*((PO_2_)_sym_long_) + *I*((PO_2_)_sym_short_) + *I*((PO_2_)_asym_) for PIG-*x*Sr and MPG-*y*Sr, respectively, where *I* denotes the amplitude of each peak. The normalized integrated intensities of the glasses are shown as a function of SrO substitution percentage in Figure 3. The integrated intensity of *Q_P_*^0^ and P-O_term_(*Q_P_*^1^) groups in PIG-*x*Sr decreased, whereas that of POP_sym_ and (PO_3_)_sym_ groups increased with an increase in SrO substitution percentage. Furthermore, the integrated intensity of TiO_4_ tetrahedra and P-O-Ti bonds increased with an increase in SrO substitution percentage, whereas that of TiO_6_ octahedra showed no significant difference. In the case of MPG-*y*Sr, the intensity of POP_sym_long_ (*Q_P_*^2^) and (PO_3_)_sym_ decreased, whereas that of (PO_2_)_sym_, P-O_term_ (*Q_P_*^1^ or *Q_P_*^2^), and TiO_4_ tetrahedra increased with an increase in SrO substitution percentage. The intensity of POP_sym_short_ (*Q_P_*^2^), (PO_2_)_asym_, and POP_sym_ (*Q_P_*^1^) showed no significant difference or a slight decreasing tendency.

**Figure 4:**
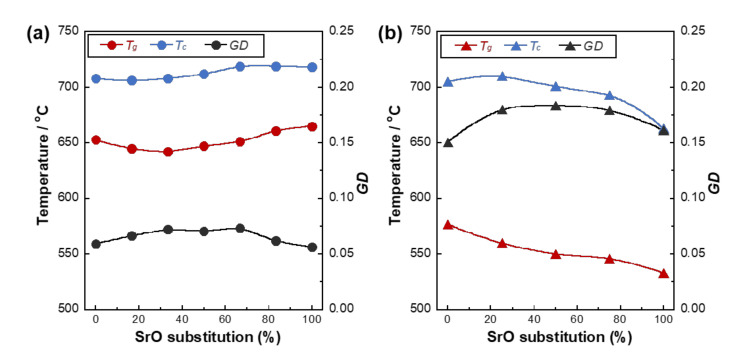
*T_g_*, *T_c_* and *GD* for (**a**) PIG-*x*Sr (circle) and (**b**) MPG-*y*Sr (triangle).

Figure 4 shows the *T_g_*, *T_c_*, and *GD* of PIG-*x*Sr and MPG-*y*Sr. The *T_g_* values of PIG-*x*Sr formed a U-shaped curve between 642 and 665 °C, with the value of PIG-33Sr as the minimum and that of PIG-100Sr as the maximum. The *T_c_* values of PIG-*x*Sr showed no significant difference or only a slight increase; the values varied from 706 to 719 °C. The *GD*s of PIG-xSr varied n-shaped curve between 0.056 and 0.074, with that of PIG-67Sr as the maximum and that of PIG-100Sr as the minimum. *T_g_* and *T_c_* values of MPG-*y*Sr showed a decreasing tendency from 577 to 533 °C and from 710 to 663 °C, respectively. The *GD*s of MPG-*y*Sr varied n-shaped curve between 0.183 and 0.151, with that of MPG-50Sr as the maximum and that of MPG-0Sr as the minimum.

**Figure 5:**
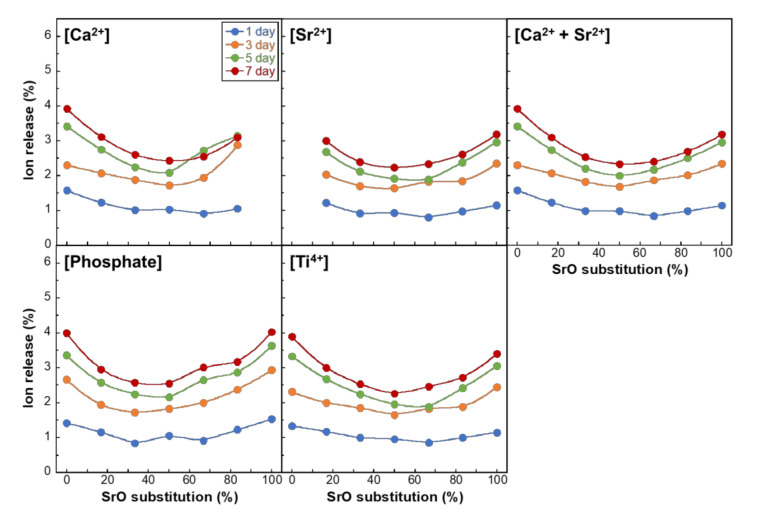
Percentage of ions release into TBS relative to the total amounts in PIG-*x*Sr for Ca^2+^, Sr^2+^, Ca^2+^ + Sr^2+^, phosphate, and Ti^4+^ ions as a function of SrO substitution percentage.

**Figure 6:**
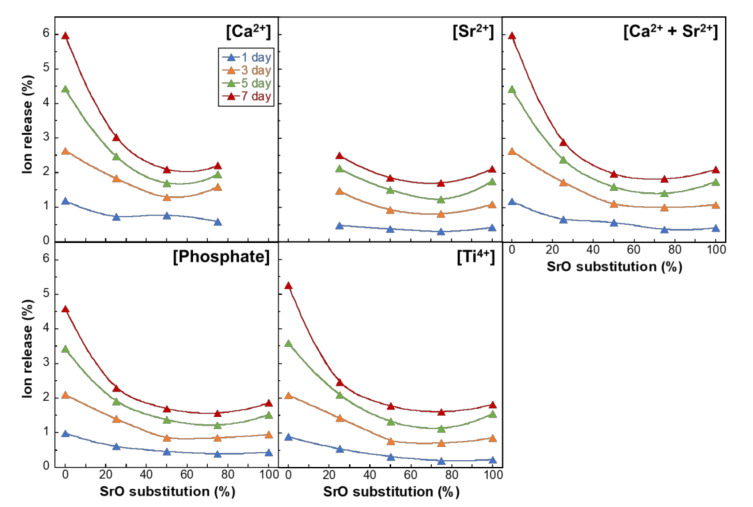
Percentage of ions release into TBS relative to the total amounts in MPG-*y*Sr for Ca^2+^, Sr^2+^, Ca^2+^ + Sr^2+^, phosphate, and Ti^4+^ ions as a function of SrO substitution percentage.

Figure 5 and Figure 6 show the ion-releasing percentages of PIG-*x*Sr and MPG-*y*Sr, respectively, into TBS, relative to the original amount in the glasses. The releasing profiles of Ca^2+^ and Sr^2+^ ions were plotted individually and considering their sum (Ca^2+^ + Sr^2+^). The ion-releasing percentages of PIG-*x*Sr resulted in U-shaped curves with an increase in SrO substitution percentage. The ion-releasing percentage of PIG-100Sr was slightly smaller than that of PIG-0Sr, and that of PIG-50Sr was the lowest. The percentages of Ca^2+^, Sr^2+^, (Ca^2+^ + Sr^2+^), phosphate, and Ti^4+^ ions at day 7 varied between 2.4% and 3.9%, 2.2% and 3.2%, 2.3% and 3.9%, 2.6% and 4.0%, and 2.3% to 3.9%, respectively. The ion-releasing behavior of MPG-*y*Sr exhibited a non-linear decreasing tendency with an increase in SrO substitution percentage. The ion-releasing percentages decreased from MPG-0Sr to MPG-50Sr and had no significant difference between MPG-50Sr, MPG-75Sr, and MPG-100Sr. The values of Ca^2+^, Sr^2+^, (Ca^2+^ + Sr^2+^), phosphate, and Ti^4+^ ions at day 7 decreased from 6.0% to 2.2%, 2.5% to 1.7%, 6.0% to 1.8%, 4.6% to 1.6%, and 5.3% to 1.6%, respectively.

## 4. Discussion

The density of PIG-*x*Sr and MPG-*y*Sr increased with an increase in SrO substitution percentage because the atomic weight of Sr (87.62) is larger than that of Ca (40.08), and the corresponding molar weight of the glass also increased with SrO substitution. However, the oxygen density of PIG-*x*Sr and MPG-*y*Sr decreased with an increase in SrO substitution percentage. The oxygen density, which is an index of the compactness of the glass network structure [40,41], of PIG-*x*Sr and MPG-*y*Sr, decreased with an increase in SrO substitution percentage. The ionic radii of Sr^2+^ (0.127 nm) is larger than that of Ca^2+^ ions (0.106 nm), and the corresponding ionic distances between oxygen ion are 0.248 and 0.269 nm, respectively [52]. Thus, the larger Sr^2+^ ions expanded the glass network, and the oxygen density of PIG-*x*Sr and MPG-*y*Sr decreased with the substitution of CaO by SrO. The rate of change in density and oxygen density of PIG-*x*Sr was larger than that of MPG-*y*Sr. PIG-*x*Sr is classified as ‘invert glass’ which contains a larger amount of network modifiers than network formers in their composition [53], whereas phosphate groups are composed of short phosphate chains, such as *Q_P_*^0^ and *Q_P_*^1^ [24]. Invert glasses did not have a continuous random network, such as a chain structure, and consisted of glass-forming tetrahedra and modifier ions linked through their non-bridging oxygen (NBO) [53]. Thus, the influence of SrO substitution on the glass network structure of PIG-*x*Sr was more than that on the structure of MPG-*y*Sr. Consequently, PIG-*x*Sr exhibited higher changing rates of density and oxygen density than MPG-*y*Sr.

The phosphate group peaks of PIG-*x*Sr and MPG-*y*Sr were red-shifted when the SrO substitution percentage increased. The bonding strength between the cation and oxygen in the glass network structure may be explained roughly by the field strength (*F*), given by the following equation [52]:

(4) $$F=\;\frac{Z_{c}}{d^{2}}\;\left(valance/{Å}^{2}\right)$$

where $Z_{c}$ is the valence of the cation and *d* is the interatomic distance between the cation and oxygen ions. The field strength of strontium and calcium are 0.28 and 0.33 valence/Å^2^, respectively. Accordingly, the bonding strength of Sr-O is weaker than that of Ca-O. Phosphate glasses are composed of phosphate groups and cations as network formers and modifiers, respectively. The cations, i.e., Ca^2+^ and Sr^2+^ ions, in PIG-*x*Sr and MPG-*y*Sr are coordinated with phosphate groups to form the glass network structure. Raman peaks of phosphate groups in phosphate glasses were red-shifted on increasing the atomic number of alkaline earth metals [54] owing to a decrease in the bonding strength between phosphate and cations [55]. Thus, the peaks of the phosphate groups in PIG-*x*Sr and MPG-*y*Sr were red-shifted on increasing the SrO substitution percentage owing to the weaker bonding strength of Sr^2+^ compared to that of Ca^2+^ ions. Similarly, the peaks of TiO_6_ octahedra and P-O-Ti bonds in PIG-*x*Sr and TiO_4_ tetrahedra in MPG-*y*Sr exhibited a red-shift with an increase in SrO substitution percentage. In contrast, the peaks of TiO_4_ tetrahedra in PIG-*x*Sr showed no significant difference. The Raman peak positions were associated species of cations around the target groups [54], as discussed previously. Hence, the network structure around the TiO_4_ tetrahedra in PIG-*x*Sr did not change with SrO substitution.

The integrated intensities of *Q_P_*^0^ ((PO_4_)_sym_) and terminal *Q_P_*^1^ decreased, whereas those of *Q_P_*^1^ (POP_sym_, (PO_3_)_sym_), TiO_4_ tetrahedra, and P-O-Ti bonds increased with increasing SrO substitution percentage in PIG-*x*Sr. The component rate in the glass can be assumed with integrated intensities of the Raman peaks [25]. Thus, phosphate groups in PIG-*x*Sr exhibited decreasing and increasing tendencies for *Q_P_*^0^ and *Q_P_*^1^, respectively. However, the terminal *Q_P_*^1^ group (P-O_term_) exhibited a decreasing tendency. This may be caused by Ti-O groups in PIG-*x*Sr. In our previous work, TiO_4_ tetrahedra acted as a network former and formed P-O-Ti bonds in phosphate invert glasses [25,56]. In addition, terminal *Q_P_*^1^ groups preferentially bond with TiO_4_ tetrahedra in phosphate invert glasses [56,57]. The peak of TiO_4_ tetrahedra in PIG-*x*Sr exhibited no shift with SrO substitution. This indicates that the TiO_4_ tetrahedra coordinated and/or bonded preferentially to the *Q_P_*^1^ group, regardless of the SrO content in the glass. Accordingly, P-O-Ti bonds in PIG-*x*Sr increased by the bond between the terminal *Q_P_*^1^ group and TiO_4_ tetrahedra. Accordingly, the integrated intensity of P-O_term_ exhibited a decreasing tendency with increasing SrO substitution percentage. The representative structural change in PIG-*x*Sr with SrO substitution is an increasing component of *Q_P_*^1^ and P-O-Ti bonds.

Raman peaks of *Q_P_*^2^ groups corresponding to POP and (PO_2_)_sym_ in MPG-*y*Sr were deconvoluted with long and short chains [51]. The long chain of *Q_P_*^2^ groups decreased with increasing SrO substitution percentage, whereas the short chain exhibited an opposite trend. Thus, the phosphate chains (*Q_P_*^2^ group) in MPG-*y*Sr were broken from long to short chains. Accordingly, the NBO and terminal *Q_P_*^2^ group exhibited an increasing tendency with SrO substitution. Strontium and calcium in metaphosphate glasses exhibit coordination numbers of 7.8 and 6.6, respectively, based on the results of structural simulation [58]. These cations are coordinated with NBO in the phosphate group, and strontium requires more NBO than calcium in the glass network structure. Thus, the shortening of the *Q_P_*^2^ chain length may have originated from the larger coordination number of strontium in metaphosphate glasses. The TiO_4_ tetrahedra group in MPG-*y*Sr increased with SrO substitution. TiO_4_ tetrahedra have been reported to act as a network former in phosphate glasses [25,59,60]. Thus, phosphate groups in MPG-*y*Sr were strongly crosslinked and/or coordinated by TiO_4_ tetrahedra groups. The POP_sym_ group in *Q_P_*^1^ of MPG-*y*Sr exhibited little change with SrO substitution, and NBO in *Q_P_*^1^ decreased. This was caused by the increased TiO_4_ tetrahedra groups in MPG-*y*Sr after SrO substitution; the group may be coordinated preferentially with NBO in the *Q_P_*^1^ group. The representative structural change in MPG-*y*Sr with SrO substitution is the shortening of the *Q_P_*^2^ chain length and crosslinking and/or coordinating of phosphate groups with TiO_4_ tetrahedra.

*T_g_* and *T_c_* are the temperatures at which the molecules begin to move and crystallize, respectively, and these temperatures are related to the glass network structure. The *T_g_* and *T_c_* values of PIG-0Sr were smaller values than those of PIG-100Sr. *Q_P_*^1^ groups and P-O-Ti bonds increased with SrO substitution in PIG-*x*Sr, as discussed previously. Hence, the glass network structure of PIG-100Sr was more crosslinked with P-O-Ti bonds, as its *T_g_* and *T_c_* values were larger than those of PIG-0Sr. MPG-*y*Sr exhibited opposite trends compared with PIG-*x*Sr, where the *T_g_* and *T_c_* values of MPG-0Sr were larger than those of MPG-100Sr. The *Q_P_*^2^ group of MPG-*y*Sr was transformed from a long chain to a short chain by SrO substitution, as mentioned above. Thus, the smaller *T_g_* and *T_c_* values of MPG-100Sr are related to the comparably short *Q_P_*^2^ chain structure of the glass, which may require less energy for the movement and crystallization of the molecules.

The *GD*s of PIG-100Sr and MPG-100Sr are expected to have smaller values than those of PIG-0Sr and MPG-0Sr as Sr^2+^ ions exhibit smaller field strength than Ca^2+^ ions, i.e., the bonding of the Sr^2+^ ion strength is smaller. However, the *GD*s of PIG-100Sr and PIG-0Sr exhibited similar values, whereas that of MPG-100Sr was larger than that of MPG-0Sr. This was owing to the corresponding glass network structures. The amount of P-O-Ti bonds in PIG-100Sr is larger than that in PIG-0Sr, and the bond is expected to improve *GD* owing to the crosslinking of phosphate groups. Substituted Sr^2+^ ion induced a decrease in *GD* of PIG-*x*Sr, because phosphate glasses with a short phosphate chain, i.e., invert glasses, formed their network structure via interactions between modifier ions and phosphate groups [61]. Consequently, the *GD* of PIG-100Sr was similar to that of PIG-0Sr because the value presumably increased as the P-O-Ti bonds increased and decreased as the SrO substitution percentage increased. The phosphate chain group of MPG-100Sr was coordinated with TiO_4_ tetrahedra, which can act as a network former. As a result, the *GD* of MPG-100Sr was larger than that of MPG-0Sr. The *GD*s of PIG-*x*Sr and MPG-*y*Sr were approximately 0.06 and 0.16, respectively. Ouchetto et al. reported that *GD*s of metaphosphate glasses and invert glasses were approximately 0.20 and 0.07, respectively [42]. Additionally, *GD*s of our previous phosphate invert glasses were 0.05~0.08 [25,27,29,30]. Thus, the *GDs* of PIG-*x*Sr and MPG-*y*Sr were in good agreement with previous reports of invert and metaphosphate glasses, respectively.

*T_g_* and *GD* for PIG-*x*Sr and *T_c_* and *GD* for MPG-*y*Sr exhibited non-linear trends as a function of SrO substitution percentage. PIG-*x*Sr and MPG-*y*Sr were prepared by the substitution of CaO by SrO. Mixed cation glasses exhibited non-linear trends in their properties [41,62,63], and these trends were also observed in our previous studies on mixed cation glasses [26,29,35]. PIG-*x*Sr and MPG-*y*Sr may indicate a mixed cation effect. This effect is associated with the movement of the cation in the glass network structure [64,65]. Smaller ions strongly reduce their mobility by weakly polarized oxygens that belong to larger ions [64]. In mixed cation glass, the cations in the glass network structure are located at energetically favorable sites [65], and the ions block each other’s pathway for migration [66]; As a result, the ions require energy for their movement. Thus, non-linear trends in the thermal properties of PIG-*x*Sr and MPG-*y*Sr were believed to be due to the mixed cation effect; similar trends were observed in our previous work on phosphate glasses with the substitution of CaO with MgO [35].

All of the ions, such as [Ca^2+^ + Sr^2+^], phosphate, Ti^4+^ ions, in the glasses released at similar rates. Thus, the glasses showed congruent dissolution, which indicated that no selective ion leaching occurred, similar to our previous phosphate glasses [25]. The field strength of Sr^2+^ ions is smaller than that of Ca^2+^ ions, and the bonding strength of cations in the glass network structure is expected to decrease with SrO substitution. As a result, the substitution of SrO with CaO may decrease the chemical durability of glasses. However, the ion-releasing percentages of PIG-100Sr and MPG-100Sr were smaller than those of PIG-0Sr and MPG-0Sr, respectively. PIG-*x*Sr and MPG-*y*Sr contain TiO_2_ in their composition, and the components are reported to improve the chemical durability of phosphate glasses [23,67]. Additionally, the P-O-Ti bonds in the PIG-*x*Sr and TiO_4_ tetrahedra in MPG-*y*Sr increased with SrO substitution. Thus, the structural changes of titanate groups in PIG-*x*Sr and MPG-*y*Sr with SrO substitution improved the chemical durability by crosslinking and/or coordinating with the phosphate groups. The SrO/CaO mixed compositions of PIG-*x*Sr and MPG-*y*Sr exhibited non-linear trends in ion-releasing behavior, which may indicate the mixed cation effect. The glasses with mixed cation compositions exhibited larger chemical durability than single cation glasses [62,63]. In our previous works on phosphate glasses, the chemical durability of mixed cation compositions was larger than that of single cation compositions [29,30,35]. In PIG-*x*Sr and MPG-*y*Sr, the chemical durability improved by structural changes in the titanium group, and SrO/CaO-mixed compositions exhibited the mixed cation effect on ion-releasing behavior. Barbara et al. reported that 0.1~1 mM of Sr^2+^ ion exhibited stimulation of ALP activity and collagen synthesis in MC3T3-E1 cells [12]. In our previous MgO-CaO-SrO-P_2_O_5_-TiO_2_ invert glasses exhibited stimulation of ALP activity in MC3T3-E1 cells, where the glasses indicated Sr^2+^ ion releasing amount of above 0.1 mM at day 7 [30]. Sr^2+^ ion releasing amount of PIG-83Sr and -100Sr was larger than 0.1 mM at day 7, whereas the amount of MPG-100Sr was 0.04 mM. Thus, PIG-83Sr and -100Sr would be expected to enhance bone formation by stimulation of ALP activity.

## 5. Conclusions

The structure and dissolution behaviors of quaternary CaO-SrO-P_2_O_5_-TiO_2_ glasses with pyro- and meta-phosphate compositions and CaO substituted by SrO were investigated. The substitution of CaO by SrO may weaken the glass network structure in PIG-*x*Sr and MPG-*y*Sr glasses because the field strength of Sr^2+^ ions is lower than that of Ca^2+^ ions. However, *GD* and chemical durability of PIG-100Sr and MPG-100Sr were improved compared with those of PIG-0Sr and MPG-0Sr, respectively. The titanium groups in PIG-*x*Sr and MPG-*y*Sr were closely related to their glass network structures by SrO substitution. Furthermore, the P-O-Ti bonds in PIG-*x*Sr and TiO_4_ tetrahedra in MPG-*y*Sr increased with substitution by SrO. These structural changes induced improvements in *GD* and chemical durability of PIG-100Sr and MPG-100Sr. The SrO/CaO-mixed compositions of PIG-*x*Sr and MPG-*y*Sr exhibited the mixed cation effect on *GD* and ion-releasing behavior, which showed non-linear trends as a function of SrO substitution percentage. These glasses may be suitable candidates for controlled Sr^2+^, Ca^2+^, and phosphate ion-releasing biomaterials on efficient design of their glass network structures.

## Figures and Tables

**Table 1:** Nominal compositions in mol% of the glasses and glass code.

Glass Code	CaO	SrO	P_2_O_5_	TiO_2_	SrO for CaO Substitution (%)
Phosphate invert glass series					
PIG-0Sr	60	-	30	10	0
PIG-17Sr	50	10	30	10	16.7
PIG-33Sr	40	20	30	10	33.3
PIG-50Sr	30	30	30	10	50.0
PIG-67Sr	20	40	30	10	66.7
PIG-83Sr	10	50	30	10	83.3
PIG-100Sr	-	60	30	10	100
Metaphosphate glass series					
MPG-0Sr	45	-	50	5	0
MPG-25Sr	33.75	11.25	50	5	25
MPG-50Sr	22.5	22.5	50	5	50
MPG-75Sr	11.25	33.75	50	5	75
MPG-100Sr	-	45	50	5	100

**Table 2:** Raman band assignments for PIG-*x*Sr and MPG-*y*Sr.

Raman Shift/cm^−1^	Assignments
585	P-O symmetric stretching vibration mode of *Q_P_*^0^
640	Ti-O stretching vibration mode of TiO_6_ octahedra
695	POP symmetric stretching mode of bridging oxygen(*Q_P_*^2^ long chain)
740	POP symmetric stretching mode of bridging oxygen(*Q_P_*^2^ short chain)
745, 770	POP symmetric stretching mode of bridging oxygen (*Q_P_*^1^)
890	Ti-O stretching vibration mode of TiO_4_ tetrahedra
945	PO_4_ symmetric stretching mode of non-bridging oxygen (*Q_P_*^0^)
990	P-O-Ti bonds
1005, 1035	PO_3_ symmetric stretching mode of non-bridging oxygen (*Q_P_*^1^)
1095	P-O vibration mode of the terminal for phosphate chains
1125	P-O stretching mode of the terminal *Q_P_*^1^
1150	PO_2_ symmetric stretching mode of non-bridging oxygen(Q_P_^2^ long chain)
1170	PO_2_ symmetric stretching mode of non-bridging oxygen(Q_P_^2^ short chain)
1265	PO_2_ asymmetric stretching mode of non-bridging oxygen (*Q_P_*^2^)

## Data Availability

Data sharing is not applicable to this article.
